# Towards a Vaccine Against Rheumatic Fever

**DOI:** 10.1080/17402520600877026

**Published:** 2006

**Authors:** L. Guilherme, K. C. Faé, F. Higa, L. Chaves, S. E. Oshiro, S. Freschi de Barros, C. Puschel, M. A. Juliano, A. C. Tanaka, G. Spina, J. Kalil

**Affiliations:** 1 School of Medicine Heart Institute (InCor) University of São Paulo São Paulo Brazil; 2 Institute for Investigation in Immunology Millennium Institute São Paulo Brazil; 3 Federal University of São Paulo (UNIFESP) São Paulo Brazil; 4 Clinical Immunology and Allergy Department of Clinical Medicine School of Medicine University of São Paulo São Paulo Brazil

## Abstract

Rheumatic fever (RF) is an autoimmune disease which affects more than 20 million children in developing countries. It is triggered by *Streptococcus pyogenes* throat infection in untreated susceptible individuals. Carditis, the most serious manifestation of the disease, leads to severe and permanent valvular lesions, causing chronic rheumatic heart disease (RHD). We have been studying the mechanisms leading to pathological autoimmunity in RF/RHD for the last 15 years. Our studies allowed us a better understanding of the cellular and molecular pathogenesis of RHD, paving the way for the development of a safe vaccine for a post-infection autoimmune disease. We have focused on the search for protective T and B cell epitopes by testing 620 human blood samples against overlapping peptides spanning 99 residues of the C-terminal portion of the M protein, differing by one amino acid residue. We identified T and B cell epitopes with 22 and 25 amino acid residues, respectively. Although these epitopes were from different regions of the C-terminal portion of the M protein, they showed an identical core of 16 amino acid residues. Antibodies against the B cell epitope inhibited bacterial invasion/adhesion *in vitro*. Our results strongly indicated that the selected T and B cell epitopes could potentially be protective against *S. pyogenes*.


*Clinical & Developmental Immunology*, June–December 2006; 13(2–4): 125–132

Taylor & Francis

Taylor & Francis Group

### Towards a vaccine against rheumatic fever

L. GUILHERME[1,2], K. C. FAÉ[1,2], F. HIGA[1,2], L. CHAVES[1,2], S. E. OSHIRO[1,2], S. FRESCHI DE BARROS[1,2], C. PUSCHEL[1,2], M. A. JULIANO[3], A. C. TANAKA[1], G. SPINA[1], & J. KALIL[1,2,4]

[1]*School of Medicine, Heart Institute (InCor), University of São Paulo, São Paulo, Brazil,* [2]*Institute for Investigation in Immunology, Millennium Institute, São Paulo, Brazil,* [3]*Federal University of São Paulo (UNIFESP), São Paulo, Brazil, and* [4]*Clinical Immunology and Allergy, Department of Clinical Medicine, School of Medicine, University of São Paulo, São Paulo, Brazil*

**Abstract**

Rheumatic fever (RF) is an autoimmune disease which affects more than 20 million children in developing countries. It is triggered by *Streptococcus pyogenes* throat infection in untreated susceptible individuals. Carditis, the most serious manifestation of the disease, leads to severe and permanent valvular lesions, causing chronic rheumatic heart disease (RHD). We have been studying the mechanisms leading to pathological autoimmunity in RF/RHD for the last 15 years. Our studies allowed us a better understanding of the cellular and molecular pathogenesis of RHD, paving the way for the development of a safe vaccine for a post-infection autoimmune disease. We have focused on the search for protective T and B cell epitopes by testing 620 human blood samples against overlapping peptides spanning 99 residues of the C-terminal portion of the M protein, differing by one amino acid residue. We identified T and B cell epitopes with 22 and 25 amino acid residues, respectively. Although these epitopes were from different regions of the C-terminal portion of the M protein, they showed an identical core of 16 amino acid residues. Antibodies against the B cell epitope inhibited bacterial invasion/adhesion *in vitro*. Our results strongly indicated that the selected T and B cell epitopes could potentially be protective against *S. pyogenes*.

**Keywords:** *Rheumatic fever,* Streptococcus pyogenes*, T and B cell protective epitopes, vaccine*

### Introduction

Rheumatic fever (RF) is a post-infectious autoimmune disease which affects over 20 million children worldwide, most of which are from developing countries. This disease affects 3–4% of untreated susceptible individuals infected by *Streptococcus pyogenes*. Carditis is the most serious manifestation of the disease, leading to severe and permanent valvular lesions, which result in chronic rheumatic heart disease (RHD).

RHD continues to be a major public health problem in developing countries, leading to 233,000 deaths/year (Carapetis et al. 2005a,b). The incidence of RHD in the world is at least 15.6 million cases and the highest documented prevalence of the disease among children from developing countries is 5.7 per 1000 in sub-Saharan Africa (Carapetis et al. 2005a,b). Epidemiological data from many developing countries are still of poor quality, and the numbers of RHD cases are surely higher than those known. In Brazil, although the incidence of acute rheumatic fever (ARF) has decreased by 75% in the last 10 years, it is still high, reaching 5000 new cases/year (data from the Brazilian Health Ministry).

The highest incidence of ARF described is among the aboriginal communities of Northern of Australia (245-351/100,000 per year) and New Zealand (80–100/100,000 per year) with an estimated 60% of cases leading to RHD (Carapetis et al. 2005a,b).

In addition to RF and RHD, *S. pyogenes* may also cause glomerulonephritis, another poststreptococcal

Correspondence: L. Guilherme, Laboratório de Imunologia, Instituto do Coração (HC-FMUSP), Av. Dr. Eneas de Carvalho Aguiar, 44-9 andar. 05403-000 São Paulo, SP, Brazil. Tel: 55 11 3069 5901; 3082 7730. Fax: 55 11 3069 5953. E-mail: luizagui@usp.br

ISSN 1740-2522 print/ISSN 1740-2530 online © 2006 Taylor & Francis
DOI: 10.1080/17402520600877026

nonsuppurative sequela, and severe invasive infections, as well as uncomplicated pharyngites and pyoderma.

The pathogenesis of RF and RHD depends on several host factors that mediate a pathological autoimmune response triggered by a defensive response against *S. pyogenes*. Genetic predisposition is one of the factors contributing to the development of autoimmunity. Several genetic markers have been studied; however the most consistent associations were described for HLA class II genes. The HLA-DR7 allele is the one most frequently associated with the disease and seems to be related to the development of multiple valvular lesions in RHD patients (see review Guilherme et al. 2005). HLA class II molecules are expressed on the surface of antigen-presenting cells (APCs)—such as macrophages, dendritic cells, and B cells—and, together with bound peptide antigen, trigger the activation of T cells. The interaction of HLA molecules, antigenic peptide and T cell receptor (TCR) on the surface of T cells are crucial for the activation of the immune response. In autoimmune disease, HLA molecules combined with certain peptides might cause inappropriate T cell activation (Brand et al. 2005). In the case of RF, during the throat infection period, several streptococcal peptides are generated after processing by macrophages and/or dendritic cells and, in association with certain HLA class II molecules, activate $CD4^+$ T cells that trigger humoral and cellular immune responses against the bacteria that will later lead to an autoimmune response (Figure 1). In RF/RHD, the molecular mimicry mechanism mediates the process whereby T cells recognize self-antigens with some degree of homology to streptococcal antigens, and provide help to autoreactive B cells. Both antibodies and T cells play an important role in the pathogenesis of RF/RHD. Several studies of the humoral immune response allowed for the definition of three major groups of crossreactivity, involving the recognition of **1** alpha-helical coiled-coil molecules such as myosin, tropomyosin and keratin; **2** myosin and DNA; and **3** myosin and *N*-acetyl-glucosamine (reviewed by Guilherme et al. 2006). The deposition of these antibodies in the valve endothelium surface triggers the inflammatory process, facilitating T cell infiltration (Cunningham 2003). The presence of $CD4^+$T cells in the heart lesions of RHD patients was described in the 1980s (Raizada et al. 1983) and the functional role of these cells was described 12 years later, when molecular mimicry between the M protein from beta-hemolytic streptococci and heart tissue proteins was shown for the first time through the analysis of heart-tissue infiltrating T cell repertoires leading to local tissue damage in RHD (Guilherme et al. 1995).

Considering that RF/RHD are the prototypes for post-infectious autoimmune disease, our greatest challenge is to develop a vaccine to prevent *S. pyogenes* infection and the diseases caused by these bacteria without inducing autoimmune reactions.

Efforts to produce a vaccine against *S. pyogenes* date from several decades ago, and are based on the use of the M protein as an inducer of protection. The first vaccine

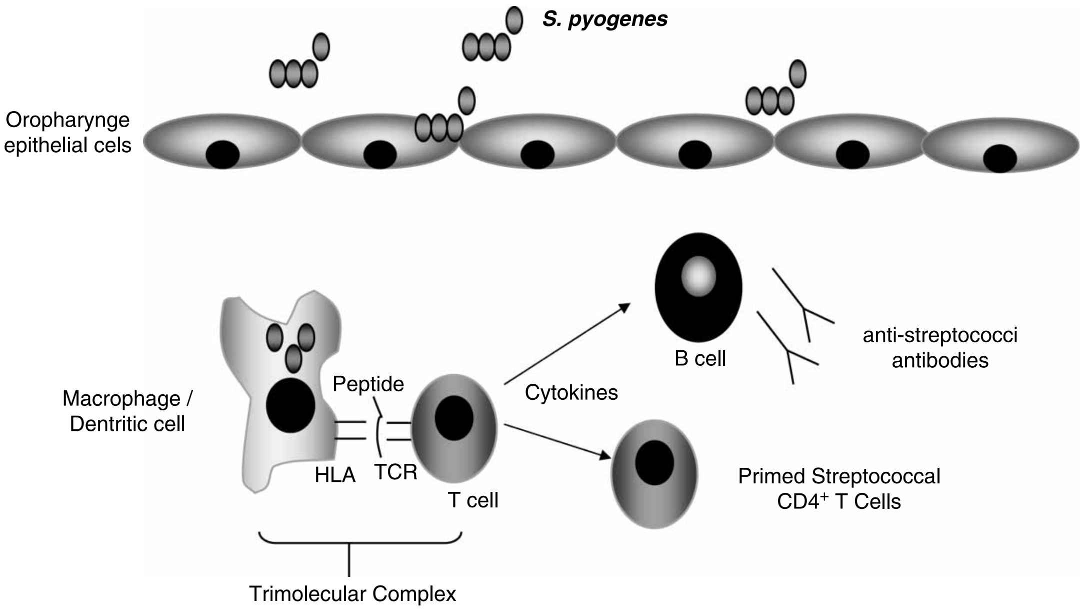

Figure 1. Schematic representation of streptococcal throat infection and the activation of the immune response. In RF, during the throat infection period, several streptococcal peptides are generated after processing by macrophages and/or dendritic cells and, in association with certain HLA class II molecules, activate $CD4^+$T cells. The complex formed by HLA molecules, antigenic peptide and TCR is called a "trimolecular complex" and is fundamental for triggering humoral and cellular immune responses to the bacteria that later may lead to an autoimmune response in susceptible individuals.

assays conducted in humans used purified streptococcal M protein and evoked protective-type specific immune responses (Polly et al. 1975; Beachey et al. 1979). Following the idea of a type-specific vaccine, Dale and co-workers developed different vaccine models using a multivalent type-specific recombinant N-terminal M protein, some of which are in clinical trial (Kotloff et al. 2004; McNeil et al. 2005).

In parallel, other groups are using the conserved C-region of the streptococcal M protein, which is common to most strains (Fischetti et al. 1988), for developing a vaccine able to provide protection against the majority of streptococcal strains. Fischetti and co-workers defined M protein C-terminal peptides capable of inducing IgA antibodies and reported the prevention of bacterial colonization in animal models (Bessen and Fischetti 1988a,b). Following these experiments, a live mucosal vaccine using *Streptococcus gordonii*, a commensal organism, engineered to express C-repeat M protein epitopes was developed and tested in mice (Medaglini et al. 1995). The use of this commensal vector as a vaccine delivery vehicle is being evaluated. Preliminary results show that the vector is safe and well tolerated when tested by oral and nasal routes in 150 healthy volunteers (Kotloff et al. 2005).

As mentioned before, RF/RHD incidence is very high among Australian and New Zealand aborigines, and, since there are particular streptococcal strains involved with disease in these countries, a vaccine model based on a combination of C- and N-terminal peptides is being assayed (Brandt et al. 2000). The most recent results from this group show that J14, a 29-mer peptide sequence which contains a conserved epitope from the C-terminal repeat of the streptococcal M protein, elicited protective opsonic antibodies against several GAS isolates. *In vivo* challenge experiments have also confirmed the protective efficacy of immunization with J14 peptide in different formulations (Vohra et al. 2005; Batzloff et al. 2005; Olive et al. 2005).

Although these various vaccine models are currently being developed, we have designed a new model of an anti-streptococcal vaccine based on the new conception of the mechanisms of autoimmune reactions leading to RF/RHD. Our studies have focused on the search for protective T and B cell epitopes by using a large panel of human blood samples, tested against overlapping peptides derived from the C-terminal portion of the streptococcal M protein differing by only one amino acid residue. Our approach takes into consideration the affinity of selected epitopes of binding to HLA class II molecules and the ability of the peptide-HLA class II complex to activate T cells via antigen TCR. T cell activation will trigger B cell immune response inducing the production of antibodies with protective potential. This strategy has allowed us to define a common T and B cell epitope that is recognized by most subjects tested. The efficacy of the vaccine epitope for inducing both humoral and cellular responses will be tested in experimental animal models as a second step in the validation of the epitope as a vaccine candidate.

### Materials and methods

### *Patients*

Blood samples from 296 patients including 129 with RF (mean age 13.8 ± 4.8) and 167 with RHD (91 patients with mean age 15.1 ± 4.7 and 76 with mean age 34.5 ± 12.0) were collected. These patients were followed for at least 5 years at the Heart Institute (InCor), HC-FMUSP, in Sao Paulo, Brazil. All patients were diagnosed according to the Jones criteria (Dajani et al. 1995). Three hundred twenty-four healthy subjects (mean age 37.7 ± 9.3) with no previous history of RF or recently documented streptococcal throat infection were also included in the study. All subjects were tested for the presence of antibodies to streptolysin O (ASLO). Blood-sample collection procedures were approved by the Heart Institute Ethics Committee, and informed consent was obtained from parents of patients under age 18 years participating in the study.

### *Peptides synthesis*

Streptococcal M5 peptides were synthesized by solid phase technology using 9-fluorenylmethoxycarbonyl (Fmoc) chemistry (Atherton and Sheppard 1989) on an automated benchtop simultaneous multiple solid-phase peptide synthesizer PSSM8 (Shimadzu, Japan) checked by mass spectrometry (Ettan Maldi-TofPro, Amershan-GE, Sweden), and purified using high-pressure liquid chromatography (RP-HPLC, Shimadzu, Japan). Seventy-nine overlapping C-terminal M5 20-mer peptides differing by only one amino acid residue, were synthesized based on the previously described streptococcal M5 protein sequence (Figure 2) (Robinson et al. 1991). In a first step, we tested the 79 overlapping peptides by ELISA using serum samples from 250 subjects, in order to select those peptides most frequently recognized. Thirty-eight overlapping peptides were selected and then the number of serum samples was increased (620 samples). The cellular response of 258 individuals was analyzed by proliferation assays to 38 selected overlapping peptides.

### *ELISA*

Ninety-six-well high binding plates (Immunoware, Pierce Biotechnology, Rockford, USA) were previously coated with streptococcal M5 peptides (25 μg/ml) in 0.05 M carbonate buffer pH 9.6 for 1 h at 37°C. After washing with 0.1% PBS-Tween buffer and blocking with 2% PBS-BSA buffer, serum

$^{253}$ SEASRKGLRRDLDASREAKKQLEAEQQK LEEQNKISEASRKGLRRDLD

ASREAKKQVEKALEEANSKLALEKLNKELEESKKLTEKEKAELQAKLEA $^{350}$

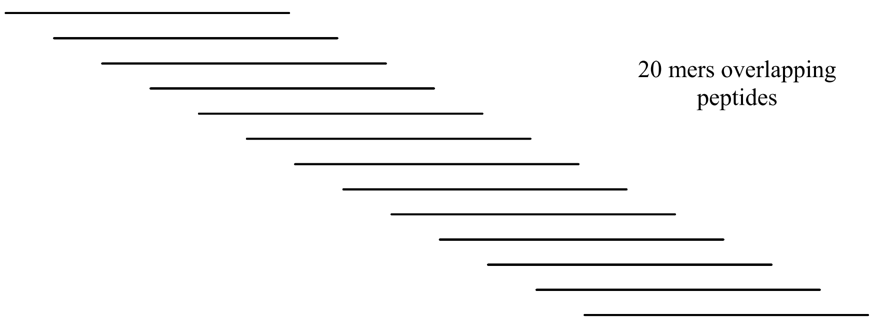

Figure 2. M protein C-terminal overlapping peptides. A region of 99 amino acid residues from the C-terminal portion of the M5 protein (residues 253–350) (Robinson et al. 1991) was covered by 79 overlapping 20-mer peptides differing by one amino acid residue.

samples (1:100 in 1% PBS-BSA buffer) were added and incubated for 18 h at 4°C. Bound antibodies were detected using a secondary antibody (peroxidase labeled rabbit anti-human IgG, Dako, Denmark) incubated for 1 h at 37°C. After washing, OPD (o-phenylediamine, Sigma-Aldrich Corporation, St Louis, Missouri, USA) substrate was added for 30 min at 37°C. The reaction was stopped with 2 M $H_2SO_4$, and M5 peptide-specific IgG was detected by reading the OD at 490 nm using an automated plate reader (Bio-Tek Instruments, Inc.—μQuant, Winooski, Vermont, USA).

*Streptococcal adhesion and invasion assay*

*Cell cultures*. HEp2 cells (human larynx carcinoma) were cultured in Dulbecco's Modified Eagle Medium (D-MEM, Invitrogen, Carlsbad, CA, USA) supplemented with L-glutamine 1 mM, gentamicin 40 μg/ml, cephalosporin 0.1 ng/ml and 10% heat-inactivated fetal calf serum (FCS), in a 5% $CO_2$ incubator. After cell expansion, 3 ml of trypsin-versene solution (0.2% trypsin, 0.2% versene in PBS pH 7.4) were added to remove the monolayer adherent cells. Cells were then transferred to 24 wells plates with a 13 mm glass slide.

*S. pyogenes strains*. *S. pyogenes* isolates were recovered from random samples of oropharyngeal, tonsillar, skin, or blood infections and other sources from patients treated at the Clinical Hospital of the University of Sao Paulo, and at the Laranjeiras National Cardiology Institute, both in Brazil. Identification was based on characteristic hemolysis in blood agar and sensitivity to bacitracin. After *emm* gene amplification and sequencing, M strains were identified in a databank (BLAST 2 (National Center for Biotechnology Information; available at http://www.ncbi.nlm.nih.gov/BLAST and CDC, Department of Health and Human Services, Centers for Disease Control and Prevention, available at http://www.cdc.gov/).

*Adhesion/invasion assay*. Semiconfluent monolayers of HEp2 cells grown on glass cover slips incubated without antibiotics in 24-well plates were infected with streptococcus suspensions ($7 \times 10^5$ bacteria) for 2 h at 37°C. Cells were then washed extensively with PBS and fixed with methanol for 20 min and stained with Giemsa for microscopy analysis. For the adherence and invasion assay infected cells were treated with trypsin-versene for 3–4 min at 37°C and lysed by addition of 0.025% Triton-X100. Cell lysates were diluted in phosphate buffered saline (PBS) and plated on blood agar for the quantification of the number of viable bacteria measured as Colony Forming Units (CFU). The ability to inhibit adhesion/invasion was assayed with a pool of diluted sera (1:30) from healthy subjects selected based on their humoral responses to C-terminal streptococcal M protein peptides. Sera were incubated with bacterial cells in suspension for 1 h at 37°C and then incubated with HEp2 cells as described above. The percentage of inhibition was calculated based on the numbers of streptococci CFU before and after sera treatment.

*Proliferation assay*. Proliferation assays were performed in 96-well plates by incubating $10^5$ PBMC separated by density gradient centrifugation ($d = 1078$ g/ml) with 10 μg/ml of M protein derived-peptides for 5 days at 37°C in a humidified 5% $CO_2$ incubator. Negative controls were PBMC and medium in the absence of peptides and positive controls were PBMC stimulated with PHA-P (2.5 μg/ml, Sigma-Aldrich Corporation, St Louis, Missouri, USA). Antigens were tested in triplicate and pulse-labeled with 0.5 μCi/well of tritiated thymidine (Amersham Pharmacia, England,

Table I. Humoral reactivity against streptococcal M protein C-terminal peptides.

| Identification | M protein C-terminal peptides amino acid residues | Reactivity (%) | |
|---|---|---|---|
| | | RF $n = 296$ | HC $n = 323$ |
| PepVac10 | $^{253}$SEASR*KGLRRDLDASREAKK*$^{272}$ | 85.5 | 92.0 |
| PepVac11 | $^{254}$EASR*KGLRRDLDASREAKKQ*$^{273}$ | 90.2 | 90.7 |
| PepVac12 | $^{255}$ASR*KGLRRDLDASREAKKQ*L$^{274}$ | 83.8 | 84.8 |
| PepVac49 | $^{291}$SR*KGLRRDLDASREAKKQ*VEKK$^{310}$ | 84.1 | 78.6 |
| PepVac50 | $^{292}$R*KGLRRDLDASREAKKQ*VEK$^{311}$ | 91.9 | 87.6 |
| PepVac51 | $^{293}$*KGLRRDLDASREAKKQ*VEKA$^{312}$ | 87.5 | 88.2 |
| PepVac18 | $^{261}$*RRDLDASREAKKQ*LEAEQQK$^{280}$ | 83.1 | 77.4 |
| PepVac38 | $^{280}$KLEEQNKISEASR*KGLRRDL*$^{299}$ | 76.7 | 81.1 |
| PepVac42 | $^{284}$QNKISEASR*KGLRRDLDASR*$^{303}$ | 81.1 | 80.8 |
| PepVac43 | $^{285}$NKISEASR*KGLRRDLDASRE*$^{304}$ | 75.3 | 80.2 |
| PepVac45 | $^{287}$ISEASR*KGLRRDLDASREAK*$^{306}$ | 76.7 | 80.8 |

RF: RF patients; HC: healthy controls. Peptides selected with more than 75% of recognition in all tested groups (healthy individuals and RF patients). Superscript numbers indicate the amino acid location in the all streptococcal M protein. A common amino acid sequence between all peptides is underlined and bold typed.

UK) in the final 18 h of culture. Cells were harvested and analysed using a beta counter (Beta plate 1205-LKB). Proliferative response was considered as positive when the stimulation index (SI) was $\geq 2.0$.

*HLA typing*. Hundred and ninety seven subjects (106 RF patients and 91 healthy controls) were typed for HLA-DRB1, DRB3, DRB4, DRB5 and DQB1 by PCR reactions using a commercial kit (micro SSP™ DNA,One Lambda, Ca, USA), following the manufacture instructions.

*Statistical analysis*. The cut-off for humoral responses tested by ELISA was established based on the OD distribution of sera from healthy individuals and RF patients, analysed using the box-plot method and the Kruskal-Wallis Test (Rosner 1986) and defined as the median of OD values of each peptide tested.

### Results

### *Definition of a B cell epitope in the C-terminal region of M protein*

Aiming to find a common streptococcal epitope able to induce the production of antibodies in healthy individuals as well as in RF patients, we evaluated the IgG response of 250 serum samples to 79 overlapping C-terminal streptococcal M protein peptides (Figure 2). Our results showed two regions of reactivity in the sequence analyzed, one region composed of 38 overlapping peptides (residues 253–317), recognized by at least 50% of subjects tested, and another region (residues 318–350), recognized by a few subjects (data not shown). After this initial screening, we increased the number of samples to 620 (324 from healthy individuals and 296 from RF patients), which were tested against the 38 selected peptides. Eleven overlapping peptides were recognized by more than 75% of both RF patients and healthy subjects (Table I), which allowed us to define a 25-residue B cell epitope (Table IV). Interestingly, when we aligned the amino acid residues of the B cell epitope, we observed a common core of 11 amino acid residues (LRRDLDASREA).

### *Sera reactive to the B cell epitope inhibited adhesion and invasion of* S. pyogenes *in vitro*

We tested the ability of two pools of sera from healthy individuals with positive and negative anti-streptolysin O (ASLO), both reactive to the selected B cell epitope, to inhibit cellular adhesion and invasion of HEp2 cells by several strains of *S. pyogenes* (M1, M5, M6, M71, M87 and M22). We observed a high percentage of inhibition of adhesion/invasion of strains M5 and M6 (99%), M44/61 (98%), M87 (97%), M1 (90%), M71 and M22 (70%) (Table II), in both pools of sera.

Table II. *S. pyogenes* adhesion/invasion inhibition assay induced by human sera.

| *S. pyogenes* strains* | Adhesion/invasion inhibition (%) |
|---|---|
| M5 | 99.0 |
| M6 | 99.0 |
| M44/61 | 98.0 |
| M87 | 97.0 |
| M1 | 90.0 |
| M71 | 70.0 |
| M22 | 70.0 |

* *S. pyogenes* strains were isolated from tonsils, throat or skin human samples (Clinical Hospital, School of Medicine, University of São Paulo, São Paulo and Laranjeiras Cardiology Institute, Rio de Janeiro, Brazil) and identified by sequencing and alignment with streptococcal strains previously described in data base (CDC, Bioedit and EMBL gene bank). Sera that inhibited the invasion were selected from healthy individuals based on their antibody reactivity against streptococcal C-terminal peptides (Table I).

Table III. Cellular reactivity against streptococcal M protein C-terminal peptides.

| Identification | M protein C-terminal peptides amino acid residues | Reactivity (%) RF $n = 133$ | Reactivity (%) HC $n = 125$ |
|---|---|---|---|
| PepVac44 | $^{286}$KISEASR*KGLRRDLDASREA* $^{305}$ | 26.2 | 26.5 |
| PepVac11 | $^{254}$EASR*KGLRRDLDASREAKKQ* $^{273}$ | 18.0 | 4.0 |
| PepVac48 | $^{290}$ASR*KGLRRDLDASREAKKQ*V$^{309}$ | 27.4 | 19.6 |
| PepVac13 | $^{256}$SR*KGLRRDLDASREAKKQ* LE$^{275}$ | 14.8 | 16.0 |
| PepVac14 | $^{257}$R*KGLRRDLDASREAKKQ* LEA$^{276}$ | 19.0 | 17.0 |
| PepVac50 | $^{292}$R*KGLRRDLDASREAKKQ*VEK$^{311}$ | 30.1 | 20.0 |
| PepVac15 | $^{258}$*KGLRRDLDASREAKKQ* LEAE$^{277}$ | 45.1 | 33.3 |
| PepVac51 | $^{293}$*KGLRRDLDASREAKKQ*VEKA$^{312}$ | 20.0 | 14.3 |
| PepVac16 | $^{259}$*GLRRDLDASREAKKQ* LEAEQQK$^{278}$ | 26.7 | 15.8 |
| PepVac52 | $^{294}$*GLRRDLDASREAKKQ*VEKAL$^{313}$ | 15.2 | 12.0 |
| PepVac17 | $^{260}$*LRRDLDASREAKKQ* LEAEQQ$^{279}$ | 21.6 | 14.3 |
| PepVac53 | $^{295}$*LRRDLDASREAKKQ*VEKALE$^{313}$ | 33.0 | 18.4 |

RF: RF patients; HC: healthy controls. Peptides selected with more than 10% of recognition in at least one of the tested groups (healthy individuals and RF patients). Superscript numbers indicate the amino acid location in the all streptococcal M protein. A common amino acid sequence between all peptides is underlined and bold typed.

### *Definition of a T cell epitope in the C-terminal region of M protein*

We evaluated the cellular immune response of 258 individuals to the same 38 overlapping C-terminal M protein peptides analysed in the humoral assays. Based on the reactivity to 12 overlapping peptides (Table III), we also defined a T cell epitope composed of 22 amino acid residues (Table IV). When we aligned the overlapping peptides we found a common core composed of 11 amino acid residues (KGLRR-DLDASR).

It is interesting to note that, although located in different regions of the C-terminal portion, both T and B cell epitopes showed an identical core of 16 amino acid residues—KGLRRDLDASREAKKQ (Table IV). Once we could define both T and B epitopes we constructed an entire peptide including both regions to be tested as a vaccine candidate in animal models (ongoing experiments). The identified peptides sequences were deposited at the Brazilian Patent Office, INPI020050020064.

### *T cell epitope recognition is not HLA class II restricted*

We performed HLA-DR and DQ typing of 197 individuals (106 RF patients and 91 healthy controls) previously tested for T cell immune response. We observed that the recognition of selected peptides (Table III) was not restricted to any specific HLA-DR or DQ antigens (data not shown).

Table IV. Alignment of T and B epitopes and definition of common amino acid residues.

| Epitopes | Amino acid sequence |
|---|---|
| T cell | KGLRRDLDASREAKKQLEAEQQ |
| B cell | EASR*KGLRRDLDASREAKKQ*VEKA |

Underlined—the 16 amino acid residues common to both T and B epitopes.

### Discussion

The development of a protective immune response to prevent group A streptococci infections and RF/RHD still remains a question that requires a deep knowledge about the mechanism leading to pathological autoimmune reactions and how a vaccine could act to protect and avoid such effects. Nowadays we know that both B and T immune responses are involved in the M protein and human tissue proteins through crossreactive reactions. There are evidences that the pathogenesis of rheumatic carditis is mediated by heart tissue crossreactive antibodies that cause an inflammation into the valve endothelium that facilitates T cells infiltration (Cunningham 2003). Heart-infiltrating cells in both myocardium and valves produce inflammatory cytokines that enhance the autoimmune reactions. Additionally, the scarce numbers of cells producing the regulatory cytokine IL-4 in the valves are probably responsible for the progression and maintenance of chronic valve lesions (Guilherme et al. 2004). These heart-infiltrating T cells recognize peptides from both N-terminal region of M protein and heart-tissue derived proteins such as myosin and vimentin through molecular mimicry (Guilherme et al. 1995, 2001; Faé et al. 2006).

In the last 20 years several researchers have been striving to develop a safe and efficacious vaccine against group A streptococci (Bisno et al. 2005). As mentioned before, the challenge to produce such vaccine is to induce protection against *S. pyogenes* without developing autoimmune reactions that could trigger RF. Antibodies directed against M protein could trigger both autoimmunity and protection (Cunningham 2000 review). Vaccine strategies targeting N-terminal segment elicit type-specific antibodies and vaccines based on C-terminal region evoke broad serotype protective antibodies.

So far there is no available vaccine, however two phase I clinical trials are in progress and both are based on the N-terminal region. One of these studies evaluated the safety and immunogenicity of a recombinant group A streptococcal vaccine containing N-terminal M protein fragments from serotypes 1, 3, 5, 6, 19 and 24 and was tested in 28 healthy adult volunteers (Kotloff et al. 2004). The follow-up showed that the vaccine was well tolerated and evoked type-specific opsonic antibodies against multiple serotypes of group A streptococcus without eliciting antibodies that cross-react with host tissues. Lately, a 26-valent vaccine has been constructed that includes 80–90% of serotypes that cause invasive infections or pharyngitis in North America (Shulman et al. 2004). This vaccine includes recombinant proteins that contain N-terminal peptides from streptococcal protective antigen and M proteins of 26 common pharyngitis, invasive, and/or rheumatogenic serotypes and was tested in 30 healthy adult volunteers (McNeil et al. 2005). However, it is important to consider that the vaccine coverage may not be the same in other continents, mainly in developing countries where there is little information regarding the distribution of M serotypes. Those results have shown that an intramuscular dose of the vaccine did not present evidence of rheumatogenicity or nephritogenicity, and did not induce the production of human tissue-reactive antibodies.

The model we propose here focused on the C-terminal portion of M protein and is similar to other groups (Brandt et al. 2000; Medaglini et al. 2005). Differently from previously proposed models, our approach searchs for a protective B and T cell epitopes using a large panel of human samples. This strategy allowed us to construct a candidate vaccine segment composed by both T and B epitopes with 16 identical amino acids. The advantage of such construction is the possibility to induce both T and B memory cells that will probably elicit a stronger protective immune response. In addition, the selected epitopes apparently were able to bind to any HLA class II molecules and activated T cells without HLA class II restriction as measured by proliferation assays.

A similar construction was done by Good's group in Austrália. They identified a region on the C-terminal portion of M protein from prevalent strains of an Aborigenal endemic area first named P145 (Pruksakorn et al. 1994; Brandt et al. 2000) that resembles the B cell epitope identified in our study along with the Brazilian population. The same group proposed a new vaccine segment called J14 by modifying their first construction (P 145) due to the observed crossreactions with myosin and keratin (Hayman et al. 1997).

In order to analyse whether the selected vaccine epitope may induce any kind of crossreactivity with heart tissue proteins we are testing the reactivity of the selected peptides with human heart- infiltrating T cell lines (HIL) obtained from RHD patients by proliferation assay and cytokine production. Our preliminary results did not show crossreactivity (ongoing experiments) indicating that the selected region could be a good candidate vaccine. The use of HIL from RHD patients is unique and reliable to control potential pathological autoimmune reactions due to the vaccine agent. Additionally, the vaccine epitope is being tested in animal models.

### Acknowledgements

This work was supported by grants from "Fundação de Amparo a Pesquisa do Estado de São Paulo (FAPESP)" $n^0$ 00/14549-4 and "Conselho Nacional de Desenvolvimento Científico e Tecnológico (CNPq)" $n^0$ 301775/83-4.

### References

Atherton e, Sheppard RC. 1989. Solid phase peptide synthesis: A practical approach. Oxford, UK: IRL Press. p 152.

Batzloff MR, Yan H, Davies MR, Hartas J, Lowell GH, White G, Burt DS, Leanderson T, Good MF. 2005. Toward the development of an antidisease, transmission-blocking intranasal vaccine for group a streptococcus. J Infect Dis 192(8):1450–1455.

Beachey EH, Stollerman GH, Johnson RH, Ofek I, Bisno AL. 1979. Human immune response to immunization with a structurally defined polypeptide fragment of streptococcal M protein. J Exp Med 150(4):862–877.

Bessen D, Fischetti VA. 1988a. Influence of intranasal immunization with synthetic peptides corresponding to conserved epitopes of M protein on mucosal colonization by group A streptococci. Infect Immun 56(10):2666–2672.

Bessen D, Fischetti VA. 1988b. Passive acquired mucosal immunity to group A streptococci by secretory immunoglobulin A. J Exp Med 167(6):1945–1950.

Bisno AL, Rubin FA, Cleary PP, Dale JB. National Institute of Allergy and Infectious DiseasesOctober 15 2005. Prospects for a group A streptococcal vaccine: Rationale, feasibility, and obstacles—report of a National Institute of allergy and infectious diseases workshop. Clin Infect Dis Oct 15;41(8):1150–1156.

Brand O, Gough S, Heward J. 2005. HLA, CTLA-4 and PTPN22: The shared genetic master-key to autoimmunity? Exp Rev Mol Med 7(23):1–15.

Brandt ER, Sriprakash KS, Hobb RI, Hayman WA, Zeng W, Batzloff MR, Jackson DC, Good MF. 2000. New multi-determinant strategy for a group A streptococcal vaccine designed for the Australian aboriginal population. Nat Med 6(4):455–459.

Carapetis JR, McDonald M, Wilson NJ. 2005a. Acute rheumatic fever. Lancet 366(9480):155–168.

Carapetis JR, Steer AC, Mulholland EK, Weber M. 2005b. The global burden of group A streptococcal diseases. Lancet Infect Dis 5(11):685–694.

Cunningham MW. 2000. Pathogenesis of group A streptococcal infections. Clin Microbiol Rev 13(3):470–511.

Cunningham MW. 2003. Autoimmunity and molecular mimicry in the pathogenesis of post-streptococcal heart disease. Front Biosci 8:s533–s543.

Dajani A, Taubert K, Ferrieri P, Peter G, Shulman S. 1995. Treatment of acute streptococcal pharyngitis and prevention of rheumatic fever: A statement for health professionals. Committee on rheumatic fever, endocarditis, and kawasaki disease of the council on cardiovascular disease in the young, the American heart association. Pediatrics 96(4 Pt 1):758–764.

Fae KC, Diefenbach da Silva D, Oshiro SE, Tanaka AC, Pomerantzeff PMA, Douay C, Charron D, Toubert A, Cunningham MW, Kalil J, Guilherme L. 2006. Mimicry in recognition of cardiac myosin peptides by heart-intralesional T cell clones from rheumatic heart disease. J Immunol 176(9):5662–5670.

Fischetti VA, Jones KF, Hollingshead SK, Scott JR. 1988. Structure, function, and genetics of streptococcal M protein. Rev Infect Dis 10(Suppl 2):S356–S359.

Guilherme L, Oshiro SE, Fae KC, Cunha-Neto E, Renesto G, Goldberg AC, Tanaka AC, Pomerantzeff PM, Kiss MH, Silva C, Guzman F, Patarroyo ME, Southwood S, Sette A, Kalil J. 2001. T-cell reactivity against streptococcal antigens in the periphery mirrors reactivity of heart-infiltrating T lymphocytes in rheumatic heart disease patients. Infect Immun 69:5345–5351.

Guilherme L, Cury P, Demarchi LM, Coelho V, Abel L, Lopez AP, Oshiro SE, Aliotti S, Cunha-Neto E, Pomerantzeff PM, Tanaka AC, Kalil J. 2004. Rheumatic heart disease: Proinflammatory cytokines play a role in the progression and maintenance of valvular lesions. Am J Pathol 165:1583–1591.

Guilherme L, Kalil J, Cunningham M. 2006. Molecular mimicry in the autoimmune pathogenesis of rheumatic heart disease. Autoimmunity 39(1):31–39.

Guilherme L, Cunha-Neto E, Coelho V, Snitcowsky R, Pillegi F, Kalil J. 1995. Human heart-infiltrating T cell clones from rheumatic heart disease patients recognize both streptococcal and cardiac proteins. Circulation 92:415–420.

Hayman WA, Brandt ER, Relf WA, Cooper J, Saul A, Good MF. 1997. Mapping the minimal murine T cell and B cell epitopes within a peptide vaccine candidate from the conserved region of the M protein of group A streptococcus. Int Immunol 9:1723–1733.

Kotloff KL, Corretti M, Palmer K, Campbell JD, Reddish MA, Hu MC, Wasserman SS, Dale JB. 2004. Safety and immunogenicity of a recombinant multivalent group a streptococcal vaccine in healthy adults: Phase 1 trial. JAMA 292(6):709–715.

Kotloff KL, Wasserman SS, Jones KF, Livio S, Hruby DE, Franke CA, Fischetti VA. 2005. Clinical and microbiological responses of volunteers to combined intranasal and oral inoculation with a *Streptococcus gordonii* carrier strain intended for future use as a group A streptococcus vaccine. Infect Immun 73(4): 2360–2366.

McNeil SA, Halperin SA, Langley JM, Smith B, Warren A, Sharratt GP, Baxendale DM, Reddish MA, Hu MC, Stroop SD, Linden J, Fries LF, Vink PE, Dale JB. 2005. Safety and immunogenicity of 26-valent group a streptococcus vaccine in healthy adult volunteers. Clin Infect Dis 41(8):1114–1122.

Medaglini D, Pozzi G, King TP, Fischetti VA. 1995. Mucosal and systemic immune responses to a recombinant protein expressed on the surface of the oral commensal bacterium *Streptococcus gordonii* after oral colonization. Proc Natl Acad Sci USA 92(15):6868–6872.

Olive C, Hsien K, Horvath A, Clair T, Yarwood P, Toth I, Good MF. 2005. Protection against group A streptococcal infection by vaccination with self-adjuvanting lipid core M protein peptides. Vaccine 23(17–18):2298–2303.

Polly SM, Waldman RH, High P, Wittner MK, Dorfman A. 1975. Protective studies with a group A streptococcal M protein vaccine. II. Challenge of volenteers after local immunization in the upper respiratory tract. J Infect Dis 131(3):217–224.

Pruksakorn S, Currie B, Brandt E, Phornphutkul C, Hunsakunachai S, Manmontri A, Robinson JH, Kehoe MA, Galbraith A, Good MF. 1994. Identification of T cell autoepitopes that cross-react with the C-terminal segment of the M protein of group A streptococci. Int Immunol 6(8):1235–1244.

Raizada V, Williams RC Jr, Chopra P, Gopinath N, Prakash K, Sharma KB, Cherian KM, Panday S, Arora R, Nigam M, Zabriskie JB, Husby G. 1983. Tissue distribution of lymphocytes in rheumatic heart valves as defined by monoclonal anti-T cell antibodies. Am J Med 74(1):90–96.

Robinson JH, Atherton MC, Goodacre JA, Pinkney M, Weightman H, Kehoe MA. 1991. Mapping T-cell epitopes in group A streptococcal type 5 M protein. Infect Immun 59:4324–4331.

Rosner B. 1986. Fundamentals of biostatistics. 2nd ed. Massachusetts: PWS publishers. p 33–35.

Shulman ST, Tanz RR, Kabat W, Kabat K, Cederlund E, Patel D, Li Z, Sakota V, Dale JB, Beall B. US Streptococcal Pharyngitis Surveillance Group 2004. Group A streptococcal pharyngitis serotype surveillance in North America, 2000–2002. Clin Infect Dis 39:325–332.

Vohra H, Dey N, Gupta S, Sharma AK, Kumar R, McMillan D, Good MF. 2005. M protein conserved region antibodies opsonise multiple strains of *Streptococcus pyogenes* with sequence variations in C-repeats. Res Microbiol 156(4):575–582.

